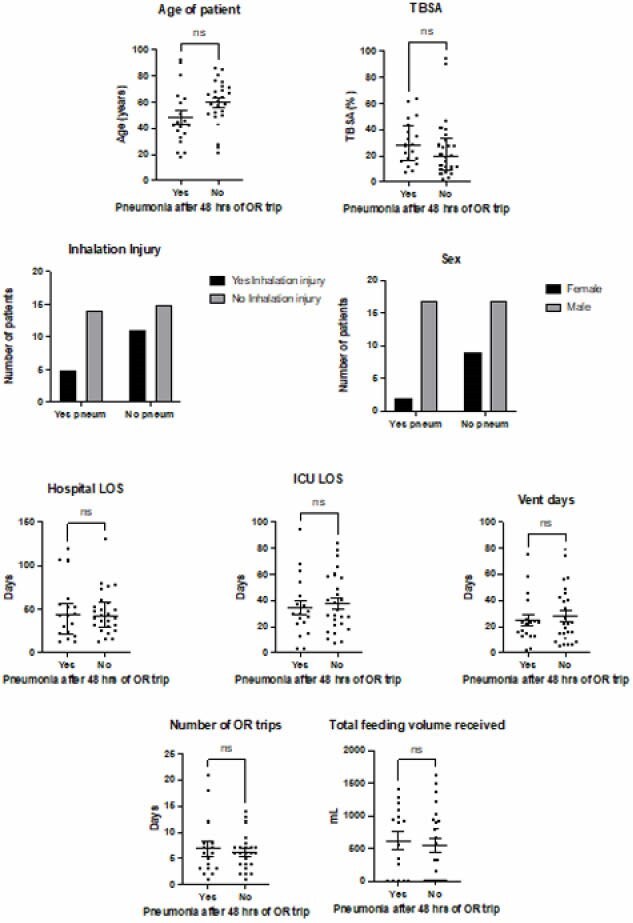# 867 Analysis of Intraoperative Gastric Feeding and Pneumonia Development

**DOI:** 10.1093/jbcr/iraf019.398

**Published:** 2025-04-01

**Authors:** Katherine Wallace, Laura Johnson, Silvia Figueiroa, Bonnie C Carney, Amber Himmler, Brynley Dean, Vivien Pat, Jeffrey Shupp

**Affiliations:** Burn Center at MedStar Washington Hospital Center; Walter L. Ingram Burn Center at Grady Memorial Hospital; Medstar Washington Hospital Center; Medstar Washington Hospital Center; Harbor University of California Los Angeles Medical Center; Inova Health Systems; Medstar Washington Hospital Center; Medstar Washington Hospital Center

## Abstract

**Introduction:**

A common barrier to meeting nutrition needs in burn patients is frequent operating room (OR) procedures. For those patients with a protected airway not requiring prone position, it is standard practice at our facility to provide tube feeding via nasogastric tube throughout operations. The purpose of this study was to review if post-operative pneumonia (PNA) development was associated with intraoperative feeding.

**Methods:**

A retrospective chart review of adult burn patients admitted to an American Burn Association (ABA) verified burn center from 2019-2021 with at least 10% total body surface area (TBSA) burn was performed. A total of 45 patients were evaluated. All patients were fed via gastric access.

The patients were grouped by those that had a positive PNA diagnosis within 48 hours of their first trip to the OR and those that did not. Continuous variables were assessed for normality using the Kolmogrov-Smirnov test. Normally distributed data were expressed as mean+/- SEM and the two groups were compared using un-paired students t-tests. Non-normally distributed data were expressed as median (IQR) and the two groups were compared using Mann-Whitney tests. Categorical variables were expressed as number (percentage) and the two groups were compared using Chi square tests.

**Results:**

Development of PNA within 48 hours of OR occurred for 19 patients (42.2%). The additional 26 patients did not develop PNA within 48 hours of OR (57.8%). There were no statistically significant differences between groups in percentage of TBSA (p=0.13), presence of inhalation injury (p=0.35), age (p=0.05), sex (p=0.08), hospital length of stay (LOS) (p=0.78), intensive care unit LOS (p=0.65), ventilator days (p=0.83), or total number of OR procedures (p=0.89). Total feeding volume received on OR day was also not significantly different between the groups (p=0.50).

**Conclusions:**

In a group of patients fed during the OR via gastric access, no significant differences in outcomes were noted between those that developed PNA and those that did not.

**Applicability of Research to Practice:**

A variety of methods are utilized to attempt to adequately meet nutrition needs in burn patients, this study highlights a strategy that could be considered at other institutions.

**Funding for the Study:**

N/A